# Primate brain architecture and selection in relation to sex

**DOI:** 10.1186/1741-7007-5-20

**Published:** 2007-05-10

**Authors:** Patrik Lindenfors, Charles L Nunn, Robert A Barton

**Affiliations:** 1 Department of Zoology, Stockholm University, S-106 91 Stockholm, Sweden; 2 Max Planck Institute for Evolutionary Anthropology, 04103 Leipzig, Germany; 3 Department of Integrative Biology, University of California, Berkeley, CA 94720-3140, USA; 4 Evolutionary Anthropology Research Group, Durham University, Durham DH1 3HN, UK; 5 PO Box 194 23, 202 KNH, Nairobi, Kenya

## Abstract

**Background:**

Social and competitive demands often differ between the sexes in mammals. These differing demands should be expected to produce variation in the relative sizes of various brain structures. Sexual selection on males can be predicted to influence brain components handling sensory-motor skills that are important for physical competition or neural pathways involving aggression. Conversely, because female fitness is more closely linked to ecological factors and social interactions that enable better acquisition of resources, social selection on females should select for brain components important for navigating social networks. Sexual and social selection acting on one sex could produce sexual dimorphism in brain structures, which would result in larger species averages for those same brain structures. Alternatively, sex-specific selection pressures could produce correlated effects in the other sex, resulting in larger brain structures for both males and females of a species. Data are presently unavailable for the sex-specific sizes of brain structures for anthropoid primates, but under either scenario, the effects of sexual and social selection should leave a detectable signal in average sizes of brain structures for different species.

**Results:**

The degree of male intra-sexual selection was positively correlated with several structures involved in autonomic functions and sensory-motor skills, and in pathways relating to aggression and aggression control. The degree of male intra-sexual selection was not correlated with relative neocortex size, which instead was significantly positively correlated with female social group size, but negatively correlated with male group size.

**Conclusion:**

Sexual selection on males and social selection on females have exerted different effects on primate brain architecture. Species with a higher degree of male intra-sexual selection carry a neural signature of an evolutionary history centered on physical conflicts, but no traces of increased demands on sociocognitive tasks. Conversely, female sociality is indicated to have driven the evolution of socio-cognitive skills. Primate brain architecture is therefore likely to be a product of ecological and species-specific social factors as well as different sex-specific selection pressures. Our results also highlight the need for acquisition and analysis of sex-specific brain components in mammals.

## Background

Primate brain architecture has been shown to correlate with both ecological [1-3] and social [4-9] factors. Social and competitive demands often differ between the sexes, however, which should leave predictable marks in the relative sizes of key brain structures [4,8-11]. The aim of this paper was to investigate the relationship between selection in relation to sex and the evolution of brain architecture in primates, and to draw attention to this largely neglected aspect of mammalian brain evolution.

Selection in relation to sex occurs whenever any genetically influenced morphological trait or behavior increases the fitness of one sex, but not the other. One well-known instance of this process in mammals is male-male competition for sexual access to females, which has resulted in a diverse array of characters important for males in conflict situations [12,13]. In primates, for example, this type of male intra-sexual selection has been shown to result in sexual size dimorphism in traits important for male combat, such as canine teeth [14,15] and body mass [16,17].

We used phylogenetic comparative methods to investigate whether two types of selection in relation to sex-intra-sexual selection on males through male-male physical competition and social selection in females-have had predictable effects on species-typical brain structures in primates. To be successful in male-male physical competition, a primate male in a polygynous species not only needs to be large and have imposing canines, but also to display, control and use his weapons and size effectively. No matter what their physical equipment might be, males without such abilities should self-evidently be less able to succeed in competitive situations. A male can potentially be proficient in the use of his assets through quick, agile and skillful movements, thus gaining an edge on his opponent physically, or he can use intelligence to outwit the competition, thus decreasing the amount of direct physical conflict necessary. The former strategy involves physical movements, while the latter involves cognitive processes intended at avoiding such movements.

From this reasoning, we derived four, not mutually exclusive, hypotheses involving male intra-sexual selection. (i) If physical combat is important, the effects of sexual selection on brain architecture should mainly act on brain regions with roles in motor skills and the coordination between sensory and motor information [10], whereas (ii) if non-combat social skills are important, we would expect to see effects of sexual selection on brain regions with more general integrative and associative functions, such as the neocortex [4-9]. (iii) It may also be the case that larger male body mass, resulting from male intra-sexual selection, puts increasing demands on the parts of the brain that handle autonomic nervous activity. (iv) Finally, selection due to increased demands from competitive situations would also be expected to influence structures that relate to aggression, threat, fear and aggression control.

We investigated four predictions derived from these hypotheses. These are all subject to the caveat that brain functions are often distributed between many separate structures; selection for a specific function will therefore tend to change several functionally connected brain structures simultaneously [18,19]. (1) We expected sexual selection for better motor skills [10] to influence components within almost all parts of the brain that function in sensory-motor activities. These range from the motor cortex and other cortical areas in the neocortex that initiate voluntary motor activities, through the mesencephalon and diencephalon, to the cerebellum in which fine adjustments of movements are made, and further through the medulla oblongata and pons. (2) In contrast, if males are more commonly in need of strategic cognitive abilities when competing, this should mainly, if not exclusively, be reflected in the relative size of the telencephalon through an increased neocortex volume [20]. (3) If effects of sexual selection are limited to effects that are caused simply by the need to control a larger body, then this should produce relatively larger brain components involved in autonomic nervous activity, primarily the hypothalamus and the medulla. (4) Structures involved in facilitating aggressive behavior (the central gray region in the mesencephalon, the hypothalamus, and amygdala) are expected to be larger in more sexually selected species; in contrast, structures relating to controlling aggression (such as the septum) should be smaller, although this last expectation is not straightforward as it could as well be argued that more aggressive species also need to better control this aggression.

We also investigated a hypothesis related to the drivers of female reproductive success. In comparison with males, female reproductive success is more closely linked to ecological factors, including the acquisition and defense of resources and protection from predators [21-23]. That causes and consequences of sociality should be considered independently for the two sexes is also indicated by observations that separate dominance hierarchies are often maintained for males and females in primate groups [24], and that primate social groups simply tend to have more females than males [25,26]. Although males also form alliances in primates [27-29], the conditions favoring these alliances tend to be more restrictive both within and across species [30,31]. Thus, cooperation is likely to be more important for females and also more closely tied to ecological demands than in males. In terms of social selection, we therefore focused on one key prediction (prediction 5): we expected that brain components involved in tasks relating to sociality and cooperation are selected for primarily in females [5,11,32], predicting that these brain structures are larger in species characterized by greater female sociality. Such brain centers are primarily, but not exclusively, found in the telencephalon (cerebrum), and within the telencephalon, in the neocortex in particular [33,34].

In summary, at least two sex-specific selection factors should leave imprints on how the brain is organized in different primate species, producing five predictions. Predictions 1–4 involve sexual selection on males for increased sensory-motor coordination, increased size of the areas relating to autonomic functions, cognitive abilities in the context of strategic planning and social networks, and changes in the structures that relate to aggression and fear. The fifth and final prediction involves social selection on females, related to increased cognitive abilities in the context of social networks.

If social or sexual selection exists, it could have one of two effects on sex-specific brain measurements. On the one hand, it could select for larger brain structures in both sexes; on the other hand, it could lead to sexual dimorphisms. Regarding the first possibility, recent research has shown that in anthropoid primates, the degree of sexual selection on males is correlated with larger canine size and greater body mass not only in males, but also in females [15,17]. Effects on females as a result of selection on males could be due to genetic correlations between the sexes in genes determining the character in question [35-39], but this is usually expected to be a temporary phenomenon [36,40,41]. More probable is that selection on females correlates with intrasexual selection operating in males [40,42], although other mechanisms are also possible [40].

Alternatively, if a selection pressure results in sexual dimorphisms in brain architecture, this would also result in a higher average value for the trait in a species, simply because the values for one sex would be increased relative to the other sex and would therefore increase the mean for that species. We therefore had strong reasons to expect that selection in relation to sex should be clearly detectable regardless of its specific effects on male and female brains. We emphasize these points because the brain volume data examined in our study are from unsexed primate specimens (Additional files 1 and 2) [43]. The results presented below provide a strong argument for obtaining sex-specific measures for more detailed analyses within and across species.

## Results

Our first set of analyses concerned the major subdivisions of the brain. These tests showed that body mass dimorphism was significantly positively correlated with the relative volumes of the medulla oblongata, mesencephalon and diencephalon, and negatively correlated with the relative volumes of the pons and telencephalon (Table 1). The telencephalon may also have been the target of social selection differing between the sexes, as indicated by a negative correlation between relative telencephalon volume and male group size, but a positive correlation with female group size, whereas correlations in the opposite direction were found in analyses of the diencephalon (Table 1). These analyses controlled for total brain volume (see Methods).

To further assess how functional differences between males and females operate on different brain structures, we analyzed specific structures of the telencephalon. The results concerning social selection showed that sociality for both males and females was correlated with different components of the telencephalon. Whereas male group size was significantly negatively correlated with the relative volumes of the septum, schizocortex and perhaps the neocortex (partial regression *p *= 0.064), female group size was positively correlated with relative neocortex volume [32] and negatively correlated with relative hippocampus volume (Table 2). Body mass dimorphism also correlated negatively with the relative sizes of the septum, striatum and schizocortex, but positively with the relative size of the amygdala (Table 2). As in the case of the brain components, these analyses also controlled for total brain volume.

For methodological reasons involving a possibly confounding effect of body mass (see Methods), we re-ran all analyses with female body mass forced into the regression models (Additional files 3 and 4). These results support the same patterns as those presented above, except that the relative volume of the pons in this scenario was not significantly correlated with sexual size dimorphism (Additional file 3). Although these results are similar to those presented, Variance Inflation Factors (VIFs) indicate that these regression models were unstable (see Methods).

To ensure that our results were not due to undue influences of results concerning the large volume of the neocortex, we repeated our analyses after removing the neocortex volumes from the "remaining brain volume" variable used to correct for allometric effects (Additional files 5 and 6). These results also support the general patterns presented above, except that the diencephalon was no longer significantly correlated with male and female group sizes, and female group size was positively correlated with the relative volumes of the septum and striatum but not significantly correlated with that of the hippocampus.

Variation in female group sizes is larger than variation in male group sizes, which could unduly influence our model choice in the stepwise regression analyses. The larger variance of female group size could tend to include female group size in the model first, thereby possibly forcing the correlated measure of male group size out of the models. For this reason, we checked all results by including the group sizes of the two sexes independently in all models. This produced qualitatively similar results to those presented above; for example, whenever male group size was non-significant when female group size was also included, it was also non-significant when female group size was not included. Thus, with only the few exceptions outlined in the previous two paragraphs, the results presented in Tables 1 and 2 remained consistent when running the analyses with alternative assumptions.

## Discussion

Our analyses indicate that selection in relation to sex has been an important influence on primate brain architecture. The results showed that sexual selection on males has acted positively on the relative sizes of the medulla, mesencephalon and diencephalon, but negatively on the pons and telencephalon. In the case of social selection, the average group size of females was positively correlated with the relative size of the telencephalon. As has already been shown elsewhere using the same dataset [32], this latter effect is apparently mediated through a positive correlation between female group size and the relative size of the neocortex. This indicates that female sociality is responsible for the evolutionary change in relative neocortex size that has taken place in haplorhine primates. These results also suggest that social demands on females and competitive demands on males require skills mainly handled by different brain components.

Our analyses fail to support the hypothesis that sexual selection on males has selected for enhanced cognitive abilities, based in particular on the negative correlation between sexual dimorphism and relative telencephalon volume, and the lack of a significant association between sexual dimorphism and neocortex volume. Further indications that selection on physical combat skills are more important is that the mesencephalon, diencephalon (containing the hypothalamus) and amygdala, all involved in governing aggressive behaviors, are positively correlated with the degree of sexual selection, whereas the septum, which has a role in facilitating aggression control, is instead negatively correlated with the degree of sexual selection. Moreover, male group size is positively correlated with the relative volume of the diencephalon (but see Additional file 5) and negatively correlated with relative septum size, further strengthening the conclusion that aggression is an evolutionarily important component of male-male interactions.

The main structures of the brain that were positively correlated with the degree of male intra-sexual competition (the medulla oblongata, mesencephalon and diencephalon) all contain important motor centers. The same is true for the cerebellum and the telencephalon, but the former is not significantly correlated with size dimorphism and the latter even exhibits a negative correlation. Given the distributed nature of the motor centers in the brain, analyses of the main brain structures provides only indirect support for the hypothesis that sexual selection acts on motor centers; more detailed data on specific brain structures are therefore needed.

The medulla oblongata and the diencephalon are important for autonomic nervous system activity. Significant correlations involving these structures suggest that larger body size resulting from sexual selection has placed demands on brain structures involved in handling a larger body. Thus, while not providing direct support, our results are in line with two hypotheses that deserve further scrutiny: that sexual selection selects for (i) brain structures important for success in male-male conflict through better body control, and (ii) better handling of a larger body through increased importance of structures involved in autonomic tasks.

The effects of social selection are more clear-cut in that they suggest that different strategies used by males and females have left marks on primate brain architecture, with female social group size positively and male group size negatively correlated with telencephalon size. Thus, demands of male and female sociality differ fundamentally in skills governed by the telencephalon, and within this region, especially the neocortex. The neocortex is important for cognitive skills involved in navigating complex webs of social relationships [44,45]. As stated in the Background, there are several reasons to expect that such social abilities concern the reproductive success of females more than males. Our results also indicate that the relationship between neocortex volume and male group size actually may be negative, further highlighting the specifically female aspect of handling social relationships in primates. More detailed analyses on the relative sizes of structures within the neocortex would be expected to reveal that it is these areas that process social information that are positively correlated with female group size.

An important question concerns the epigenetic mechanisms by which species differences in brain architecture arise during ontogeny, in particular the relative roles of early, genetically guided ontogenetic processes versus later processes influenced substantially by environmental input and/or hormones. For example, experimental evidence indicates that perturbations of sensory inputs cause neural reorganization [46,47]. However, regional differentiation occurs early in ontogeny, prior to and/or independently of neural innervation from the periphery [48,49], and mutations that influence architectonics prior to innervation by the periphery have been discovered [50]. The role of neurogenesis later in mammalian ontogeny (e.g. in adults) appears to be relatively minor in primates, and restricted to the hippocampus and olfactory bulbs [51-53]. Thus, adult neurogenesis is highly unlikely to explain species differences in overall brain architecture. Finally, many species differences in brain structure size fall outside the range of intra-species variation [18,54]. Thus, while earlier, predominantly prenatal processes are undoubtedly crucial, species differences, such as those analyzed here, may be somewhat moderated by later ontogenetic effects.

From our study of unsexed specimens, we cannot determine if social and sexual selection have produced sexual dimorphisms in brain size, or whether selection acting on one sex has had correlated effects on the other sex, thus producing larger brains for that species. The results presented here therefore emphasize the value of obtaining sex-specific data on brain structures. Several authors have proposed or tested hypotheses for sexual dimorphism in brain structures [10,11,20,55,56]. Several additional hypotheses can be tested. For example, in more social species, females should be expected to have relatively larger neocortices than males. Contrary to our results, however, the fact that reported incidences of innovation behavior are higher in males than in females across species [57] could be taken to indicate that males have greater cognitive abilities. It can possibly be argued that female primates use their neocortices mainly to navigate social webs, whereas male neocortices are free to perform other tasks. On the other hand, as indicated by our results, in more sexually selected species, a suite of other brain structures should be larger in males than in females. To investigate such sexual dimorphisms, however, sex-specific measures of brain components are necessary.

We have no reason to expect that the patterns presented here apply only to anthropoid primates; similar patterns should be detectable in other mammal clades, provided of course that sufficient variation in sociality and/or intra-sexual competition exists. It is even possible that these factors have driven brain evolution in mammals and thus may explain differences in brain architecture among different mammal orders. This especially concerns the relatively large neocortex-a hallmark of primates and a trait indicated by this study to be a consequence of the high degree of female sociality in primates-but should also involve other structures.

## Conclusion

The results presented here indicate that selection in relation to sex is an underappreciated force in primate brain evolution. Social selection on females and sexual selection on males accounts for significant variation in primate brain architecture. Whereas female sociality is tied to increased cognitive abilities, male sociality and sexual selection on males is not. Instead, sexual selection on males has favored brain structures involved in aggression, sensory motor functions and autonomic functions. This is important because selection pressures acting on the brain have previously almost exclusively been treated as uniform in males and females. Given available data, however, it is impossible to know whether these effects lead to dimorphism or correlated effects in both sexes. Development of new datasets that make use of sexed brains will be needed to address this fundamental question.

## Methods

Data on volumes of different brain structures were gathered from the literature [43,58]. The major structures of the brain included in the analyses were the pons, medulla oblongata (including the reticular formation), cerebellum (including the brachium and the nuclei pontis), mesencephalon (excluding the reticular nucleus), diencephalon and telencephalon (cerebrum). To further investigate hypotheses regarding different substructures of the telencephalon, we also used volume information for the septum, striatum, amygdala, schizocortex (entorhinal, perirhinal and presubicular cortices), hippocampus, and neocortex (isocortical grey and underlying white matter). As noted above, information was unavailable for the sexes of the animals for the brain measurements.

We also gathered data on body mass [59] and group size [60] for the species with brain data. Female group size served as a proxy for social complexity [32], whereas sexual size dimorphism was used to measure sexual selection [17]. The number of data points limited our choice for alternative variables indicating strength of sexual selection. Instead, we repeated some of the analyses using canine dimorphism as a proxy for sexual selection, and these analyses produced results similar to those for body mass. The fact that more data were available on primate body masses than canine dimensions led us to prefer the former to the latter; thus, body mass dimorphism results are presented here. Although data exist for strepsirhine primates, these were not included in the analyses because there is very little variation in both sociality and sexual size dimorphism in the species for which data on the volumes of different brain structures is also available [61-63]. All variables were log10-transformed prior to analysis.

Haplorhine (Old World) primates are generally larger, more dimorphic and live in larger groups than platyrrhine (New World) primates. That is, the causal factors we use in this study are similar within taxonomic groups, because of their shared evolutionary history. For this reason, we employed phylogenetically independent contrasts that use differences between species and taxonomic groups instead of the species' values themselves [64]. This approach produces statistics untainted by problems caused by similarity due to common descent. We used Purvis' [65] estimate of primate phylogeny, which was created using a super-tree technique to combine a large number of source phylogenies. This phylogeny uses information published to the date of its construction and unites knowledge gathered from both molecular and morphological data. It is therefore based on more information, and covers more species, than any alternative phylogeny. Hypothesis testing was performed using the aforementioned phylogenetically independent contrasts [64], as implemented in the computer program PDAP [66]. Diagnostic tests showed that branch lengths given in Purvis [64] needed no adjustment [67].

Because we were interested in investigating the effects of multiple independent variables on different brain components, we analyzed the influence of these variables using stepwise multiple regression. To investigate which variables were significantly correlated with the dependent variables, we used a backwards-removal procedure with all variables initially included in the model, and then sequentially removed variables with significance levels > 0.1. Because correlations exist between female body mass and total brain volumes, between female body mass and dimorphism, and between male and female group sizes, we tested whether collinearity rendered our multiple regression models unstable by calculating Variance Inflation Factors (VIFs) [68]. With only one exception, the VIFs were < 10, indicating that collinearity was unlikely to have a major impact on the stability of the models [68,69]. The exception involved female body mass and "remaining brain volumes," which had VIFs> 10, but these analyses were restricted to secondary analyses (Additional files 3 and 4).

To control for allometric effects, we subtracted the volume of the brain component under scrutiny from the total brain volume and used this "remaining brain volume" as a covariate in all regression models. Including total brain volume instead of the "remaining brain volume" as a covariate produced results similar to those presented here, but we feel that the measure we used better corrects for part-whole correlation problems. The volumes of all examined brain parts were closely correlated to our "remaining brain volume" measure (*p *<< 0.001; Table 1). We chose "remaining brain volume" rather than body mass when controlling for allometric effects primarily because including female body mass and "remaining brain volume" together in the regression models almost always gave non-significant partial regression coefficients for female body mass. In addition, brain volume is both statistically and conceptually closer to the brain components under scrutiny than is body mass. To make sure that our results were not due to indirect effects of body mass, we double-checked our regression models by forcing female body mass into the equations (Additional files 3 and 4). Because the effect of sexual selection on male size has been shown to be a main cause of sexual size dimorphism in haplorhine primates [17], inclusion of male body mass has the unwanted effect of including effects of sexual selection in the body mass measure. For this reason, inclusion of male body mass, or the mixed body masses of Stephan et al. [43], produced results that were difficult to interpret. Although sexual selection also affects female body mass, these effects are smaller than those on males [17].

The telencephalon (cerebrum) is by far the largest substructure in the haplorhine primate brain (65–85% of the total brain volume) and the largest substructure within the telencephalon is by far the neocortex (40–80% of the total brain volume). Selection pressures affecting the relative size of the neocortex could therefore also affect the relative sizes of all other brain components (e.g. if the neocortex becomes comparatively larger, the other brain components automatically become comparatively smaller). For this reason, we checked our results by repeating the analyses while excluding the neocortex from the "remaining brain volume" variable. Results that were statistically significant in the first round of analyses but non-significant when excluding the neocortex volumes (or *vice versa*) have to be judged carefully (Additional files 5 and 6).

## Authors' contributions

PL conceived of the hypotheses and carried out the analyses. PL, CN and RB co- wrote the paper.

## Supplementary Material

Additional File 1 Data on body mass, group size and volumes of major brain components for the primate species analyzed in this study. Click here for file

Additional File 2 Volumes of telencephalon structures for the primate species analyzed in this study. Click here for file

Additional File 3 Stepwise multiple regression models with forced inclusion of female body mass: brain components. Click here for file

Additional File 4 Stepwise multiple regression models with forced inclusion of female body mass: telencephalon components. Click here for file

Additional File 5 Stepwise multiple regression models without the neocortex: brain components. Click here for file

Additional File 6 Stepwise multiple regression models without the neocortex: telencephalon components. Click here for file

## Figures and Tables

**Table 1:** Stepwise multiple regression models: brain components

	Brain components (dependent variables)					
	
Independent variables included in the best model	Pons	Medulla oblongata	Cerebellum	Mesencephalon	Diencephalon	Telencephalon
Total brain volume minus the dependent variable	*b *= 1.233	*b *= 0.734	*b *= 1.030	*b *= 0.646	*b *= 0.841	*b *= 1.090
	*t *= 21.016	*t *= 17.239	*t *= 22.734	*t *= 20.520	*t *= 30.225	*t *= 28.424
	*p *<< 0.001	*p *<< 0.001	*p *<< 0.001	*p *<< 0.001	*p *<< 0.000	*p *<< 0.001
Sexual size dimorphism	*b *= -0.240	*b *= 0.369	-	*b *= 0.168	*b *= 0.140	*b *= -0.182
	*t *= -2.421	*t *= 5.093		*t *= 3.134	*t *= 3.294	*t *= -3.227
	*p *= 0.026	*p *<< 0.001		*p *= 0.006	*p *= 0.005	*p *= 0.005
Female group size	--	--	--	--	*b *= -0.064	*b *= 0.119
					*t *= -2.143	*t *= 3.259
					*p *= 0.048	*p *= 0.005
Male group size	--	--	--	--	*b *= 0.043	*b *= -0.062
					*t *= 2.021	*t *= -2.335
					*p *= 0.060	*p *= 0.033

Whole model	F_(2,18) _= 258.21	F_(2, 18) _= 260.89	F_(1,19) _= 516.82	F_(2,18) _= 317.32	F_(4,16) _= 409.56	F_(4,16) _= 352.48
	R^2 ^= 0.966	R^2 ^= 0.967	R^2 ^= 0.964	R^2 ^= 0.972	R^2 ^= 0.990	R^2 ^= 0.989
	*p *<< 0.001	*p *<< 0.001	*p *<< 0.001	*p *<< 0.001	*p *<< 0.001	*p *<< 0.001

The table shows results from separate multiple regression models based on independent contrasts investigating the effects of four independent variables on six different main components of the primate brain.

The models were constructed by sequentially removing variables, keeping those with *p *≤ 0.1. Each column contains one best regression model relating to that specific brain component. Numbers to the right of each independent variable are the partial regression coefficients for that specific variable, and the numbers in the bottom row give statistics for the multiple regression models. Dashes indicate variables excluded from the final best models because they had a partial regression *p *> 0.1.

**Table 2:** Stepwise multiple regression models: telencephalon components

	Telencephalon components (dependent variables)					
	
Independent variables included in the best modeL	Septum	Striatum	Amygdala	Schizocortex	Hippocampus	Neocortex
Total brain volume minus the dependent component	*b *= 0.838	*b *= 0.947	*b *= 0.581	*b *= 0.856	*b *= 0.812	*b *= 1.405
	*t *= 19.986	*t *= 18.384	*t *= 8.978	*t *= 13.085	*T *= 12.946	*t *= 21.420
	*p *<< 0.001	*p *<< 0.001	*p *<< 0.001	*p *<< 0.001	*p *<< 0.001	*p *<< 0.001
Sexual dimorphism	*b *= -0.212	*b *= -0.373	*b *= 0.363	*b *= -0.542	--	--
	*t *= -2.892	*t *= -4.258	*t *= 3.308	*t *= -4.731		
	*p *= 0.010	*p *< 0.001	*p *= 0.004	*p *< 0.001		
Female group size	--	--	--	--	*b *= -0.117	*b *= 1.136
					*T *= -2.268	*t *= 3.398
					*p *= 0.036	*p *= 0.003
Male group size	*b *= -0.071	--	--	*b *= -0.188	--	*b *= -0.058
	*t *= -3.053			*t *= -5.191		*t *= -1.984
	*p *= 0.007			*p *<< 0.001		*p *= 0.064

Whole model	F_(3,17) _= 158.25	F_(2,18) _= 182.92	F_(2,18) _= 77.256	F_(3,17) _= 67.947	F_(2,18) _= 84.643	F_(3,17) _= 409.79
	R^2 ^= 0.965	R^2 ^= 0.953	R^2 ^= 0.896	R^2 ^= 0.923	R^2 ^= 0.4907	R^2 ^= 0.986
	*p *<< 0.001	*p *<< 0.001	*p *<< 0.001	*p *<< 0.001	*p *<< 0.001	*p *<< 0.001

The table shows results from separate multiple regression models based on independent contrasts investigating the effects of four independent variables on seven different main components of the primate telencephalon.

The models were constructed by sequentially removing variables, keeping those with *p *≤ 0.1. Each column contains one best regression model relating to that specific telencephalon component. Numbers to the right of each independent variable are the partial regression coefficients for that specific variable, while the numbers in the bottom row give statistics for the multiple regression models. Dashes indicate variables excluded from the final best models because they had a partial regression *p *> 0.1.
