# Supplementary material for: Primate brain architecture and selection in relation to sex
Source: BMC Biol. 2007 May 10;5:20. doi: 10.1186/1741-7007-5-20 (PMC1885794; doi:10.1186/1741-7007-5-20)
Supplement: Additional File 1 — Data on body mass, group size and volumes of major brain components for the primate species analyzed in this study. [file 1741-7007-5-20-S1.doc]

## Table 1 - Data on body mass, group size and volumes of major brain components (mm^3^) for the primate species analyzed in this study

| Species | Male mass  (kg) | Female mass  (kg) | Number  of males | Number  of females | Total brain  volume | Pons | Medulla  oblongata | Cerebellum | Mesencephalon | Diencephalon | Telencephalon |
| --- | --- | --- | --- | --- | --- | --- | --- | --- | --- | --- | --- |
| *Callithrix jacchus* | 0.317 | 0.324 | 2.7 | 2.9 | 7241 | 38.8 | 318 | 757 | 295 | 554 | 5318 |
| *Cebuella pygmaea* | 0.11 | 0.122 | 1.5 | 1 | 4302 | 17.5 | 201 | 468 | 192 | 342 | 3099 |
| *Saguinus oedipus* | 0.418 | 0.404 | 2.6 | 1.9 | 9537 | 50.9 | 413 | 984 | 333 | 754 | 7052 |
| *Callimico goeldii* | 0.499 | 0.468 | 1 | 2 | 10510 | 53.8 | 460 | 1240 | 340 | 739 | 7733 |
| *Saimiri sciureus* | 0.899 | 0.68 | 2.7 | 7.9 | 22572 | 135.2 | 722 | 2260 | 526 | 1430 | 17635 |
| *Aotus trivirgatus* | 0.813 | 0.736 | 1.1 | 1 | 16195 | 112.2 | 686 | 1873 | 409 | 1098 | 12128 |
| *Callicebus moloch* | 1.02 | 0.956 | 1 | 1 | 17944 | 105.9 | 787 | 1622 | 530 | 1375 | 13465 |
| *Ateles geoffroyi* | 7.78 | 7.29 | 4 | 17 | 101034 | 812.8 | 1834 | 12438 | 1482 | 5334 | 79946 |
| *Lagothrix lagothricha* | 7.28 | 7.02 | 5.8 | 8.2 | 95503 | 726.1 | 2110 | 11268 | 1582 | 5721 | 74822 |
| *Macaca mulatta* | 9.355 | 7.085 | 9.1 | 23.8 | 87896 | 639.7 | 1992 | 8965 | 1380 | 4480 | 71080 |
| *Cercocebus albigena* | 8.25 | 6.02 | 4.8 | 7.8 | 97603 | 873 | 2708 | 10726 | 1770 | 5351 | 77079 |
| *Papio anubis* | 23.15 | 12.5 | 9.1 | 17.5 | 190957 | 2177.7 | 5297 | 18683 | 2711 | 9280 | 154987 |
| *Cercopithecus ascanius* | 3.7 | 2.92 | 1 | 8.5 | 63505 | 520 | 1632 | 5828 | 1162 | 3605 | 51279 |
| *Cercopithecus mitis* | 7.697 | 4.363 | 1.1 | 8.8 | 70564 | 517.6 | 1999 | 6758 | 1354 | 4176 | 56277 |
| *Erythrocebus patas* | 12.4 | 6.5 | 1.2 | 10.4 | 103167 | 814.7 | 2616 | 8738 | 1621 | 5423 | 84770 |
| *Miopithecus talapoin* | 1.94 | 1.56 | 13 | 21.3 | 37776 | 273.5 | 1035 | 3374 | 826 | 2375 | 30166 |
| *Colobus badius* | 8.36 | 8.21 | 4.4 | 11.4 | 73818 | 647.1 | 2007 | 8648 | 1333 | 3945 | 57885 |
| *Nasalis larvatus* | 20.4 | 9.82 | 1 | 3.7 | 92797 | 968.3 | 2945 | 12113 | 1556 | 5310 | 70873 |
| *Hylobates lar* | 5.9 | 5.34 | 1 | 1 | 97505 | 1166.8 | 2251 | 12078 | 1459 | 5716 | 76001 |
| *Pan troglodytes* | 49.567 | 40.367 | 6.7 | 12.4 | 382103 | 3990.2 | 5817 | 43663 | 3739 | 15392 | 313493 |
| *Gorilla gorilla* | 169.367 | 80 | 1.8 | 4.4 | 470359 | 5395.1 | 7509 | 69249 | 4352 | 19370 | 369878 |
