# Supplementary material for: Primate brain architecture and selection in relation to sex
Source: BMC Biol. 2007 May 10;5:20. doi: 10.1186/1741-7007-5-20 (PMC1885794; doi:10.1186/1741-7007-5-20)
Supplement: Additional File 2 — Volumes of telencephalon structures for the primate species analyzed in this study. [file 1741-7007-5-20-S2.doc]

## Table 2. Volumes of telencephalon structures (mm^3^) for the primate species analyzed in this study

| Species | Septum | Striatum | Amygdala | Schizocortex | Hippocampus | Neocortex |
| --- | --- | --- | --- | --- | --- | --- |
| *Callithrix jacchus* | 49.8 | 372 | 106.2 | 90 | 221 | 4371 |
| *Cebuella pygmaea* | 29.2 | 174 | 75.3 | 81.8 | 133 | 2535 |
| *Saguinus oedipus* | 60.5 | 453 | 141.9 | 107 | 262 | 5894 |
| *Callimico goeldii* | 63.5 | 493 | 146.9 | 138 | 281 | 6476 |
| *Saimiri sciureus* | 90.6 | 1042 | 242.7 | 168 | 352 | 15541 |
| *Aotus trivirgatus* | 83.8 | 862 | 193.6 | 243 | 539 | 9950 |
| *Callicebus moloch* | 86.4 | 920 | 254.1 | 234 | 588 | 11163 |
| *Ateles geoffroyi* | 324 | 4950 | 869 | 732 | 1366 | 70856 |
| *Lagothrix lagothricha* | 266 | 4947 | 753.4 | 680 | 1586 | 65873 |
| *Macaca mulatta* | 271 | 4032 | 677.6 | 639 | 1353 | 63482 |
| *Cercocebus albigena* | 294 | 4146 | 781.6 | 630 | 1485 | 68733 |
| *Papio anubis* | 559 | 7182 | 952.8 | 1309 | 3398 | 140142 |
| *Cercopithecus ascanius* | 251 | 2827 | 572.8 | 694 | 1189 | 45166 |
| *Cercopithecus mitis* | 246 | 2733 | 704.7 | 617 | 1366 | 49933 |
| *Erythrocebus patas* | 330 | 3624 | 688.7 | 693 | 1591 | 77141 |
| *Miopithecus talapoin* | 133 | 1908 | 413 | 259 | 705 | 26427 |
| *Colobus badius* | 288 | 3217 | 501.2 | 814 | 1671 | 50906 |
| *Nasalis larvatus* | 333 | 3735 | 719.4 | 855 | 1966 | 62685 |
| *Hylobates lar* | 302 | 4784 | 666.8 | 1136 | 2673 | 65800 |
| *Pan troglodytes* | 851 | 12246 | 1422.3 | 2018 | 3779 | 291592 |
| *Gorilla gorilla* | 1173 | 14567 | 2754.2 | 2729 | 4781 | 341444 |
