# Supplementary material for: Primate brain architecture and selection in relation to sex
Source: BMC Biol. 2007 May 10;5:20. doi: 10.1186/1741-7007-5-20 (PMC1885794; doi:10.1186/1741-7007-5-20)
Supplement: Additional File 3 — Stepwise multiple regression models with forced inclusion of female body mass: brain components. [file 1741-7007-5-20-S3.doc]

## Table 3 - Stepwise multiple regression models with forced inclusion of female body mass: brain components

|  | Brain Components (Dependent Variables) | | | | | |
| --- | --- | --- | --- | --- | --- | --- |
| Independent  variables included  in the best model | *Pons* | *Medulla*  *oblongata* | *Cerebellum* | *Mesencephalon* | *Diencephalon* | *Telencephalon* |
| Female body mass | b = -0.273  t = -2.665  p = 0.016 | b = 0.035  t = 0.360  p =0.723 | b = 0.207  t = 2.687  p = 0.015 | b = 0.178  t = 3.186  p = 0.005 | b = 0.039  t = 0.713  p = 0.487 | b = -0.127  t = -1.671  p = 0.115 |
| Total brain volume minus  the dependent variable | b = 1.546  t = 10.221  p << 0.001 | b = 0.692  t = 5.450  p << 0.001 | b = 0.742  t = 6.489  p << 0.001 | b = 0.447  t = 5.441  p << 0.001 | b = 0.793  t = 10.927  p << 0.000 | b = 1.257  t = 11.572  p << 0.001 |
| Sexual size  dimorphism | – | b = 0.348  t = 3.746  p = 0.002 | – | – | b = 0.116  t = 2.142  p = 0.049 | b = -0.134  t = -2.199  p = 0.044 |
| Female  group size | – | – | – | – | b = -0.064  t = -2.111  p = 0.052 | b = 0.132  t = 3.724  p = 0.002 |
| Male  group size | – | – | – | – | b = 0.042  t = 1.922  p = 0.074 | b = -0.064  t = -2.566  p = 0.022 |
| Whole  model | F_(2,18)_ = 272.12  R² = 0.968  p << 0.001 | F_(317)_ = 165.56  R² = 0.967  p << 0.001 | F_(2,18)_ = 346.63  R² = 0.975  p << 0.001 | F_(2,18)_ = 321.21  R² = 0.973  p << 0.001 | F_(5,15)_ = 317.68  R² = 0.991  p << 0.001 | F_(5,15)_ = 314.13  R² = 0.990  p << 0.001 |

The table shows results from separate multiple regression models based on independent contrasts investigating the effects of five independent variables on six different main components of the primate brain. The models were constructed by sequentially removing variables, keeping those with p≤0.1, while forcing the inclusion of female body mass. Each column contains one best regression model relating to that specific brain component. Numbers to the right of each independent variable are the partial regression coefficients for that specific variable, while the numbers in the bottom row indicate the regression coefficients for that specific multiple regression model. Dashes indicate variables excluded from the final best models because they had a partial regression p>0.1. Note that even though these regression models are similar to those given in the main text, calculated Variance Inflation Factors (VIFs) indicate that the forced inclusion of female body mass could cause the regression models to be unstable.
