# Supplementary material for: Primate brain architecture and selection in relation to sex
Source: BMC Biol. 2007 May 10;5:20. doi: 10.1186/1741-7007-5-20 (PMC1885794; doi:10.1186/1741-7007-5-20)
Supplement: Additional File 6 — Stepwise multiple regression models without the neocortex: telencephalon components. [file 1741-7007-5-20-S6.doc]

## Table 6. Stepwise multiple regression models without the neocortex: telencephalon components

|  | Telencephalon Components (Dependent Variables) | | | | |
| --- | --- | --- | --- | --- | --- |
| Independent  variables included  in the best model | *Septum* | *Striatum* | *Amygdala* | *Schizocortex* | *Hippocampus* |
| Total brain volume minus  the dependent variable  and the neocortex | b = 0.895  t = 17.427  p << 0.001 | b = 1.054  t = 17.987  p << 0.001 | b = 0.674  t = 10.419  p << 0.001 | b = 0.981  t = 13.874  p << 0.001 | b = 0.872  t = 13.146  p << 0.001 |
| Sexual  dimorphism | b = -0.249  t = -3.441  p = 0.003 | b = -0.454  t = -5.116  p = 0.0001 | b = 0.314  t = 3.176  p = 0.005 | b = -0.616  t = -5.551  p < 0.001 | – |
| Female  group size | b = 0.118  t = 2.458*  p = 0.026 | b = 0.082  t = 2.226*  p = 0.040 | – | – | –* |
| Male  group size | b = -0.130  t = -3.718  p = 0.002 | – | – | b = -0.184  t = -5.382  p < 0.001 | – |
| Whole model | F_(4,16)_ = 127.44  R² = 0.970  p << 0.001 | F_(3,17)_ = 138.02  R² = 0.960  p << 0.001 | F_(2,18)_ = 101.70  R² = 0.919  p << 0.001 | F_(3,17)_ = 76.268  R² = 0.931  p << 0.001 | F_(1,19)_ = 172.83  R² = 0.901  p << 0.001 |

The table shows results from separate multiple regression models based on independent contrasts investigating the effects of four independent variables on five different main components of the primate telencephalon. The models were constructed by sequentially removing variables, keeping those with p≤0.1. For these analyses, neocortex volume was excluded from the remaining brain volume variable. Each column contains one best regression model relating to that specific telencephalon component. Numbers to the right of each independent variable are the partial regression coefficients for that specific variable, while the numbers in the bottom row indicate the regression coefficients for that specific multiple regression model. Dashes indicate variables excluded from the final best models because they had a partial regression p>0.1. A star (*) indicates where removing neocortex volume from the remaining brain volume variable has changed a result from significant to non-significant, or vice-versa.
